# Supplementary material for: Food Waste Compost as a Tool of Microbiome-Assisted Agri-Culture for Sustainable Protection of Vegetable Crops Against Soil-Borne Parasites
Source: Int J Mol Sci. 2025 Oct 31;26(21):10606. doi: 10.3390/ijms262110606 (PMC12607853; doi:10.3390/ijms262110606)
Supplement: Supplementary file 1 [file ijms-26-10606-s001.zip › Table S1.pdf]

Table S1 Effect of low FWC1 doses on RKN-inoculated tomato plants. Plant growth was detected as shoot height (SH in cm), shoot weight (SW in g), and root weight (RW, in g); infection factors were detected as egg masses per g root fresh weight (EMs g^{-1} rfw) and sedentary forms per g root fresh weight (SFs g^{-1}), female fecundity (FF), and reproduction potential (RP). All the parameters of treated plants were referred to those of plants left untreated, as controls (Cntr). Significant changes, according to a *t*-test (P<0.05), are indicated by an asterisk. Significant difference in treated with respect to control plants (Cntr) is indicated in %.

	Cntr*	FWC1*	Cntr**	FWC1**
SH	46±9	46±8	36±6	35±9
SW	17.1±6.8	17.9±11.8	7.2±3.3	9.1±3.8* (20)
RW	1.6±1.2	1.6±1.5	1.4±0.6	1.7±0.7
EMs g^{-1}	24±16	18±8	41±22	43±21
SFs g^{-1}	61±40	49±32	80±28	63±34* (-21)
FF	183±78	243±134	--	--
RP	28±12	29±6	--	--

* juvenile plants (1.5-3.0 g); ** seedlings (5.0-7.0 g)

*FWC1 10 g kg^{-1} soil;** FWC1 5 g kg^{-1} soil
