# Supplementary material for: Food Waste Compost as a Tool of Microbiome-Assisted Agri-Culture for Sustainable Protection of Vegetable Crops Against Soil-Borne Parasites
Source: Int J Mol Sci. 2025 Oct 31;26(21):10606. doi: 10.3390/ijms262110606 (PMC12607853; doi:10.3390/ijms262110606)
Supplement: Supplementary file 1 [file ijms-26-10606-s001.zip › Table S2.pdf]

Table S2. Effect of a high dose of FWC1 (15 g kg^{-1} soil), on tomato seedlings (2.0 g at treatment). Plant growth was detected 50 days after treatment as shoot height (SH in cm), shoot weight (SW in g), and root weight (RW, in g); infection factors were detected 50 days after inoculation as egg masses per g root fresh weight (EMs g^{-1} rfw), Significant changes, according to a t-*test* (P<0.05), are indicated by are indicated by an asterisk. Significant difference in treated with respect to control plants (Cntr) is indicated in %.

	**Cntr**	**FWC1**
SH	27±5	24±6
SW	6.6±1.8	5.2±2.9* (-22)
RW	1.0±0.4	0.7±0.3* (-24)
EMs g^{-1}	77±45	42±13* (-46)
