# Supplementary material for: Food Waste Compost as a Tool of Microbiome-Assisted Agri-Culture for Sustainable Protection of Vegetable Crops Against Soil-Borne Parasites
Source: Int J Mol Sci. 2025 Oct 31;26(21):10606. doi: 10.3390/ijms262110606 (PMC12607853; doi:10.3390/ijms262110606)
Supplement: Supplementary file 1 [file ijms-26-10606-s001.zip › Table S3.pdf]

**Table S3.** Effect of a high dose of FWC1 (10 g/kg soil), on pepper seedlings. Plant growth was detected 50 days after treatment as shoot height (SH in cm), shoot weight (SW in g), and root weight (RW, in g); infection factors were detected 50 days after inoculation as egg masses per g root fresh weight (EMs $g^{-1}$ rfw), sedentary forms per g root fresh weight (SFs $g^{-1}$), female fecundity (FF), and reproduction potential (RP). Significant changes, according to a Duncan test ($P<0.05$), are indicated by by an asterisk. Significant difference in treated with respect to control plants (Cntr) is indicated in %.

| | **Cntr** | **FWC1** |
|---|---|---|
| SH | 29±11 | 29±13 |
| SW | 4.3±1.3 | 3.8±1.3. |
| RW | 1.4±0.4 | 1.2±0.4 |
| EMs $g^{-1}$ | 149±53 | 101±65* (-32) |
| SFs $g^{-1}$ | 292±130 | 247±79 |
| FF | 482±209[a] | 394±82 |
| RP | 168±22[a] | 128±16* (-24) |
