# Supplementary material for: Food Waste Compost as a Tool of Microbiome-Assisted Agri-Culture for Sustainable Protection of Vegetable Crops Against Soil-Borne Parasites
Source: Int J Mol Sci. 2025 Oct 31;26(21):10606. doi: 10.3390/ijms262110606 (PMC12607853; doi:10.3390/ijms262110606)
Supplement: Supplementary file 1 [file ijms-26-10606-s001.zip › Table S4.pdf]

Table S4. Defense-related genes examined in this study and the specific primers used in quantitative reverse transcriptase- polymerase chain reaction (qRT-PCR).

Gene acronym	Primer sequence (5'-3')
SlGPX	F: GTTTGCTTGCACACGGTTTA
	R: CGTCGTTGGTGGATACCTCT
SlPR-4b/P2	F: TGACCAACACAGGAACAGGA
	R: GCCCAATCCATTAGTGTCCA
SlCAT2	F: TGCTCCAAAGTGTGCTCATC
	R: TTGCATCCTCCTCTGAAACC
ACT-7	F: CAGCAGATGTGGATCTCAAA
	R: CTGTGGACAATGGAAGGAC
